# The psychometric evaluation of the sense of belonging instrument (SOBI) with Iranian older adults

**DOI:** 10.1186/s12877-021-02115-y

**Published:** 2021-03-29

**Authors:** Kelly-Ann Allen, Gökmen Arslan, Heather Craig, Sedigheh Arefi, Ameneh Yaghoobzadeh, Hamid Sharif Nia

**Affiliations:** 1 Educational Psychology and Inclusion, Faculty of Education, Monash University, Clayton, Australia; 2 Centre for Positive Psychology, Melbourne Graduate School of Education, Melbourne University, Melbourne, Australia; 3 Burdur Mehmet Akif Ersoy University, Burdur, Turkey; 4 International Network on Personal Meaning, Toronto, Ontario Canada; 5 School of Public Health and Preventative Medicine, Monash University, Melbourne, Australia; 6 Health Education and Health Promotion, Faculty of Medical Sciences, Tarbiat Modares University, Tehran, Iran; 7 School of Nursing and Midwifery, Tehran University of Medical Sciences, Tehran, Iran; 8 School of Nursing and Midwifery Amol, Mazandaran University of Medical Sciences, Sari, Iran

**Keywords:** Sense of belonging, Psychometric, Older adult, Aging, Belonging

## Abstract

**Background:**

A sense of belonging is a significant predictor of mental health and well-being in later life. A sense of belonging in childhood and adolescence contributes to a number of adult behavioural and psychological outcomes. A high sense of belonging has been associated with better health, longevity, psychological well-being, and disease recovery.

**Methods:**

In this study, the Persian version of the Sense of Belonging Instrument (SOBI) for older adults in Iran was evaluated psychometrically to develop an accurate measure for belonging. Participants in the study were 302 older adults, 60 years old and above, living independently in Iran and chosen through convenience sampling.

**Results:**

An exploratory factor analysis indicated that the four-factor structure, which included 16 items, accounted for 54.12% of the total variance, and was characterized by strong factor loadings, with values ranging from .50 to .87. Thereafter, a confirmatory factor analysis confirmed the four-factor latent structure of the SOBI, providing adequate data-model fit statistics. All latent structures were characterized by adequate-to-strong latent construct (H) internal reliability (α) coefficients.

**Conclusions:**

The Persian version of the SOBI is a useful tool in understanding older adult patients’ sense of belonging when living independently within the community. The implications for practice and research are discussed.

According to Hagerty et al. (1992), a sense of belonging occurs when people feel like they are an integral part of a system or environment [1]. Hagerty and colleagues proposed two characteristics that define a sense of belonging in their *Theory of Human Relatedness* (1993) [2]: 1. Valued involvement: an individual’s perception that they are valued, needed, or important in terms of other people, groups, objects, organizations, environments, or spiritual dimensions; and 2. Fit: an individual’s feeling that they *fit,* or are congruent with others, groups, organizations, environments, or spiritual dimensions [1]. Both characteristics highlight that belongingness is subjective and unique to the individual in a particular context [2, 3]. Although a sense of belonging is a distinct construct, it is commonly associated with social connectedness and negatively correlated to ostracism, loneliness, and social isolation [4–7]. However, belonging is unique because it does not always involve the presence of other people [2, 8]. For example, people may feel a sense of belonging to an environment or place [9, 10]. It is this feature of being a part of a system or environment that sets belonging apart from other similar constructs that more specifically deal with social relationships [5, 11–14].

The outcomes of a sense of belonging, according to Hagerty et al. (1992), include meaningful engagement at the psychological, social, spiritual, or physical level. A low sense of belonging is a significant predictor of mental health problems (e.g., depression and anxiety) and is related to stress, loneliness, and suicide [15–18]. In older adults specifically, research evidence has suggested that a low sense of belonging is associated with depression, suicidal ideation, and hopelessness [19–21].

The benefits of belonging include adaptive physiological and psychological outcomes such as lowered heart rate and improved social self-esteem [22]. In older adults, a sense of belonging has been associated with overall life satisfaction [23]. Bailey and McLaren (2005) found that a sense of belonging was an important protective factor for the mental health of retirees. Interventions that have sought to increase a sense of belonging in nursing home residents in Iran have found associations with enhanced mental and social health and functioning [24]. Group belonging has been found to be important for mental health, buffering the effects of depression in 60- to 75-year-olds living in Iran and Iranian immigrants living in Sweden [25].

## The implications of belonging for preventative medicine for older persons

The maintenance and promotion of mental health and well-being in later life are an important, yet an under-researched, area of knowledge [26–28]. A sense of belonging is important in older adults, with increased age being associated with a greater incidence of social isolation and loneliness [29–31], which relates to a heightened risk of health problems, hospitalization, impaired cognitive function, and mortality [32]. For many older individuals, retirement can be a time of change in social networks, personal and social identity, and time spent connecting with others. However, levels of belonging can be lower and mental health negatively affected when retired older adults lack the motivation and desire to feel a sense of belonging or are without the necessary skills to belong [10, 19].

Social groups have been found to be a protective factor for premature death in older adults who have retired [33]. Older adults are at particular risk of suicide, with the rate of suicide in older adults being the highest of any age group in almost all countries [34]. According to Kissane and McLaren (2006), a sense of belonging is associated with providing older adults a reason for living. Therefore, a better understanding of a sense of belonging in this population is warranted [35]. In a study of Veterans [36], it was found that suicidal ideation was reduced in those with meaningful social connections.

Belonging has been identified as enhancing the mental health of older residents in long-term care [37]. Belonging research for older populations has presented compelling benefits. A greater sense of belonging is associated with better health, longevity, improved psychological well-being, and quicker recovery following disease [38]. Rinnan et al. (2011) investigated joy of life in older adult residents living in nursing homes. Sense of belonging was found to be one of the core dimensions to an individual’s perception of joy in their life for this age group [39]. As for older people living independently, other research reports that a greater sense of belonging is an important protective factor against frailty. Haslam et al. (2008) demonstrated similar findings with stroke survivors. Those who reported belonging to multiple group memberships prior to their initial stroke reported better recovery outcomes. Participants who belonged to groups were more resilient to the effects associated with stroke and had a greater sense of well-being [40]. A sense of belonging in older age appears to have both psychological and physical health benefits. To make belonging a priority for older adults’ health and well-being, robust culturally specific assessment tools are needed to understand belonging in this age group, which may inform prevention and early intervention programs and provide a means to measure their efficacy in future research and practice.

### Belonging is contextual

From a research perspective, our understanding of belonging has been weighted towards literature from first world, Western countries. However, the experience of belonging is highly contextual and interplays with notions of culture, race, religion, language, and socio-historical backgrounds [41]. For instance, definitions of belonging may vary between culture and context; how an Australian Aboriginal may contextualise belonging is very different from how someone living in India may define belonging [8]. In an extensive study of 379 community college students, Hagerty et al. (1996) found no significant relationship between measures of belonging and the age, gender, marital status, education, or ethnicity of the sample [42]. However, a majority (64%) of participants in this study were Caucasian and younger adults (mean age – 26 years). Therefore, given the importance of context in understanding belonging, we seek to explore the role of a sense of belonging in a distinct population: older adults in Iran. This target population has its own needs and motivations, which are undoubtedly influenced by Iran’s cultural norms [27, 43]. Iranian culture has strong elements of tradition and family, and promotes younger family members taking care of older aged family members [44]. Most Iranians are happy to provide care to older family members in their own homes [45]. The older adult population in Iran is highly respected by family members and valued within the community [46].

### Measures of belonging

Despite the reported importance of belonging in the literature, there are relatively few measures available to assess this construct, especially in applied settings such as clinics and aged care homes. Several belonging measures rely on a scale designed to be used only for a single study (e.g., Village, 2007) [47]. Furthermore, in keeping with the contextual nature of belonging, many scales used to measure this concept are designed for use with specific populations, such as individuals from a certain country or culture [48, 49] or school students – including the Psychological Sense of School Membership Scale (PSSM) [50].

The Sense of Belonging Instrument (SOBI) is one of the most utilised measures of belonging available. The 27-item self-report measure developed by Hagerty and Patusky (1995) consists of two distinct scales: the SOBI-P (psychological state), which measures sense of belonging in terms of valued involvement and fit in relationships, and the SOBI-A (antecedents), which examines the antecedents to a sense of belonging [15]. While the SOBI-P (made up of 18 items) has been found to be a reliable and valid measure of sense of belonging, evidence for the validity and internal consistency of the SOBI-A was not as robust [15]. Despite this, the SOBI was chosen as the most suitable measure for this study because it can be used with older adults living independently, it is readily adapted to other languages, its construct validity has been ascertained using three separate measures, and the SOBI has high internal consistency [15].

The 1995 study by Hagerty and Patusky confirmed that the SOBI was positively correlated with social support (*r* = .42) and negatively correlated with loneliness (*r* = −.76). Unlike other data-driven measures of belonging, the SOBI-P was developed from the Theory of Human Relatedness [15]. There is some evidence that the SOBI may potentially be used to discover negative correlations between a sense of belonging and depressive symptoms [51] and suicidal ideation and suicide attempts [19, 42]. Furthermore, the SOBI was deemed to have high internal consistency and be a valid and reliable measure of a sense of belonging [15].

The SOBI has been translated into Thai to study the association between sense of belonging and depression in both women and adolescents, with Cronbach alphas of 0.89 (pilot study), 0.98 (sample of Thai women), and 0.84 (sample of adolescents) [52, 53]. However, two items of the English language version of the SOBI-P had to be removed because it could not be understood by a large portion (40%) of Thai individuals [52]. With the removal of the two items, the Thai version of the SOBI has a high internal consistency.

For the SOBI to be used in Iran, research is needed to evaluate the Persian version’s psychometrics as no previous studies have examined the psychometric properties of the SOBI in an Iranian context previously. However, there has been sufficient research in Persian countries on constructs related to belonging to assume that it is a construct of relevance and value [24, 25, 54].

The current study aims to psychometrically evaluate a Persian version of the SOBI for older adults in Iran. There are several potential implications for the findings of this study. First, an empirically rigorous tool for measuring belonging has utility for psychologists and other healthcare professionals who work with older adults. Second, a context-specific measurement tool may also provide researchers with the ability to measure belonging and devise interventions more accurately. In responding to the call for examining the effect of cultural factors in explaining belonging [41] and its important role in the health and well-being of older adults, a valid and reliable tool in the context of local culture is essential. Therefore, the present study has the primary focus to assess the psychometric properties of the Persian version of the SOBI among older adults in Iran.

## Methods

### Population and settings

This study’s sample consisted of 302 older adults aged 60 years or older living independently in the community. Data were collected from public places in two urban provinces in Iran: Tehran and Qazvin (i.e., in parks and mosques). Some of the participants were classified as illiterate (i.e., unable to read), so the research team explained the study's purpose in a simple and clear language. Data collection took place in 2018. Older adults were eligible if they were willing to participate, were age 60 years or over, were fully oriented to time and place, and had the ability to communicate and answer the questionnaire’s items.

### Measures

Participants completed the questionnaire that collected data on SOBI as well as demographic information. The demographic information contained participants’ age, gender, educational and socioeconomic status, marital status, and main income sources.

The Sense of Belonging Instrument (SOBI) [9] consists of 27-items and these items are divided into two domains: 1. The SOBI-P which evaluates the psychological sense of belonging (valued involvement and fit); and 2. SOBI-A which assesses the antecedents to a sense of belonging, for instance the motivation of people have towards a sense of belonging [15]. The measure employs the use of a Likert-type scale ranging from 1 (Strongly Disagree) to 4 (Strongly Agree). The items included in the SOBI-P are negatively phrased (i.e., a score of 4 indicates a low sense of belonging), while the SOBI-A uses positively phrased items (i.e., a score of 4 represents a high sense of belonging). In a sample of 250 adult participants, 61 years of age and older, the mean score on the SOBI-P was 57.68 and a standard deviation of 9.02, while the mean score on the SOBI-A was 46.60 and a standard deviation of 6.72 [35].

Written permission was obtained from Professor Hagerty, the author of SOBI to use the measure in the present study. Then, using The World Health Organization translation protocol, the SOBI was translated into Persian, with a forward-backward technique [55]. Two English-Persian translators independently translated the measure, and an expert panel comprised from representatives of the research team and translators created a single Persian translation of the SOBI. A Persian-English translator then back-translated the Persian version of SOBI to English. This back-translated version in English was then evaluated by Professor Hagerty, who confirmed that this version was similar to the original English version of SOBI.

### Data analyses

The factor structure of the SOBI was examined using exploratory and confirmatory factor analysis. Exploratory factor analysis was first performed to explore the factor structure of the measure. The Kaiser–Meyer–Olkin (KMO) and Bartlett’s test of sphericity were used to check the sample’s appropriateness to conduct the factor analysis. Factor extraction was based on: (i) eigenvalues > 1; (ii) communalities > .3; and (iii) scree plot [56]. The EFA results were interpreted with factor loading ≥ .40, cross-loading ≥ .32, and loading on a different factor [56, 57]. After revealing the scale’s factor structure, confirmatory factor analysis was conducted using maximum likelihood estimation to confirm the latent structure of the SOBI. Appropriate model-fit statistics, and their cut off scores were used to evaluate the measurement model of the SOBI: root mean square error of approximation (RMSEA ≤ .06 for good fit and ≤ .10 for acceptable fit), comparative fit index (CFI ≥ .95 for good fit and ≥ .90 for acceptable fit), non-normed fit index (NNFI ≥ .95 for good fit and ≥ .90 for acceptable fit), and standard root mean square residual (SRMR ≤ .06 for good fit and ≤ .10 for acceptable fit [58, 59].

After examining the factor structure of the measure, reliability analyses were performed. The internal (α), latent construct (*H*), and composite reliability (CR) estimates were used to evaluate the reliability of the Persian version of SOBI, and the coefficient ≥ .70 was considered adequate [60, 61]. Additionally, discriminant and convergent validity were investigated using the average variance extracted (AVE) and maximum shared variance (MSV). Convergent validity was evaluated using AVE for each construct against the scale’s correlations with the other constructs (see Hair et al. (2014) for suggested thresholds for discriminant and convergent validity analyses) [52]. The AVE ≥ .5 suggests adequate convergent validity, and the MSV should be less than the value of AVE to meet the discriminant validity [60]. All data analyses were conducted using SPSS version 24 and LISREL version 8.8.

## Results

### Sample characteristics

Most of the participants (*n* = 182; 60.3%) were male. The age of older adults who participated in this study was 67.75 ± 6.59. The majority (*n* = 258; 85.4%) of participants were married. Table 1 presents the demographic characteristics of participants in the study.

**Table 1:** Demographic profiles of respondents

Variables	N (%) or Mean (SD)
***Sex***	
Male	182 (60.3%)
Female	120 (39.7%)
**Age**	67.75 (6.59)
***Marital Status***	
Single	6 (2%)
Married	258 (85.4%)
Divorced	5 (1.7%)
Widow	33 (10.9%)
***Educational Status***	
Illiterate	14 (4.6%)
Elementary	58 (19.2%)
Junior	66 (21.9%)
Diploma	124 (41.1%)
Collegiate	40 (13.2%)
***Present Socioeconomic Status***	
Less advantaged (less than 10 million Rials)	30 (9.9%)
Moderately advantaged (between 10 million to 30 million Rials)	254 (84.1%)
Most advantaged (more than 30 million Rials)	18 (6%)
***Main Income Resources***	
Personal	64 (21.2%)
Family	76 (25.2%)
Pension	162 (53.6%)
***Relative Visiting***	
yes	269 (89.1%)
no	33 (10.9%)
***Social support (0–10)***	3.93 (2.22)
***Religious belief (0–10)***	7.01 (2.85)

Following the assumption that the normality outcomes and factors would be inversely correlated, exploratory factor analysis was conducted using principal axis factoring with a promax rotation to explore the latent factor structure of the SOBI. Preliminary analysis results showed the 27 SOBI items had adequate KMO sampling adequacy (.80) and lack of singularity (Bartlett’s *χ*^*2*^ = 4108.89, *df* = 351, *p* < .001), supporting an exploratory factor analysis. Results of the first-factor analysis revealed seven factors with eigenvalues > 1, and these constructs explained approximately 56% of the variance. However, the pattern matrix results indicated five cross-loading items (λ > .32) and five non-loading items (λ ≤ .40), and the scree plot and parallel analysis results suggested that a four-factor latent structure would be a better fit to the data. After excluding cross and non-loading items, the factor analysis was re-run, demonstrating the four-factor structure (eigenvalues > 1), which included 16 items accounting for 54.12% of the total variance, with eigenvalues of these four factors ranging from 1.21 to 5.54. The first component of the measure comprised four items related to fitting in. The second included four items measuring social esteem. The third consisted of four items assessing collective self. The final component comprised four items related to self-awareness of older adults, see Table 2. Factor loadings of the factors were strong, with values ranging from .50 to .87, and they accounted for 31.91, 12.01, 5.70, and 4.50% of the variance, respectively.

**Table 2:** Scale Items and Factor Loadings

Items	EFA	CFA				
	λ	λ_1_	*ℓ*^2^_1_	λ_2_	*ℓ*^2^_2_	*H*
Fitting In Scale	**–**	**–**	**–**	.56	.32	.85
I am troubled by feeling like I have no place in this world.	.82	.85	.72	**–**	**–**	**–**
I feel like I observe life rather participate in it.	.87	.77	.77	**–**	**–**	**–**
I feel like a square peg trying to fit into a round hole.	.70	.62	.38	**–**	**–**	**–**
I feel left out of things.	.64	.72	.52	**–**	**–**	**–**
Social Esteem Scale	**–**	**–**	**–**	.67	.45	.85
I could disappear for days and it wouldn’t matter to my family.	.70	.74	.55	**–**	**–**	**–**
If I died tomorrow, very few people would come to my...	.85	.79	.63	**–**	**–**	**–**
I could not see or call my friends for days and it wouldn’t...	.67	.76	.57	**–**	**–**	**–**
I am not valued by or important to my friends.	.78	.76	.58	**–**	**–**	**–**
Collective Self Scale	**–**	**–**	**–**	.86	74	.82
It is important to me that I am valued or accepted by others.	.63	.47	.22	**–**	**–**	**–**
I have qualities that can be important to others.	.65	.76	.57	**–**	**–**	**–**
It is important to me that my thoughts and opinions are valued.	.75	.73	.53	**–**	**–**	**–**
Generally, other people recognize my strengths and good.	.60	.78	.60	**–**	**–**	**–**
Self-Awareness Scale	**–**	**–**	**–**	.72	.51	.83
I would describe myself as a misfit in most social situations.	.54	.64	.41	**–**	**–**	**–**
I generally feel that people accept me.	.67	.75	.57	**–**	**–**	**–**
I feel like a piece of a jig-saw puzzle that doesn’t fit into the.	.82	.76	.58	**–**	**–**	**–**
I would like to make a difference to people or things around	.50	49	.24	**–**	**–**	**–**
Overall Sense of Belonging Scale	**–**	**–**	**–**	**–**	**–**	.83

*Note. EFA* exploratory factor analyses; *CFA* confirmatory factor analysis. λ_1_ = item loadings for first-order factors; *ℓ*^2^_1_ = indicator reliability for first-order factor items; λ_2_ = first-order factor loading for second-order factor; *ℓ*^2^_2_ = indicator reliability for second-order factor indicators; *H* = latent construct reliability for first-order and second-order factors

After exploring the measure’s factor structure, a confirmatory factor analysis was conducted to confirm the four-factor latent structure of the SOBI. Results from this analysis provided adequate data-model fit statistics. The Chi-square (*χ*^2^) value was significant (*χ* ^2^ = 309.99, *df* = 98, *p* < .001), and the *χ* ^2^/*df* ratio was 3.16. The root mean square error of approximation and the standardized root mean square residual values were adequate (RMSEA = .08 [95% CI: .07–.09], SRMR = .07). In addition, comparative fit index and non-normed fit index values were .90 and higher, suggesting an adequate data-model fit (CFI = .94, NNFI = .93). All latent structures were also characterized by strong latent construct reliability coefficients (*H* range = .82–.85). Factor loadings of the scales were adequate-to-strong, ranging from .47 to .85 (see Table 2).

Descriptive statistics for the final 16-item scale indicated that both scale and its latent structures had a relatively normal distribution (skewness and kurtosis < |2|), and corrected item-total correlation coefficients, which are the Pearson’s correlation between each item, ranged between moderate and large (.43 to .73). The scale also provided adequate–to–strong internal consistency reliability coefficients (α range = .75–to–.87; see Table 3). Additionally, the measurement model’s reliability and validity were investigated using composite reliability, average variance extracted, and maximum shared variance. Findings from these analyses indicated that the scales had adequate-to-strong composite reliability (CR range = .76–.85) and discriminant validity (MSV < AVE; see Table 3). However, convergent validity results showed that the AVE scores for fitting in and social esteem subdimensions were less than .50 (AVE = .48–.45). Collective self and self-awareness subscales were provided criterion of convergent validity, and the AVE scores for both scales were greater than .50 (AVE = .55–.58).

**Table 3:** Observed Scale Characteristics for the SOBI

Scales	*M*	*SD*	Skew.	Kurt.	α	CR	AVE	MSV
Fitting In	10.55	2.27	−.43	−.19	.83	.78	.48	.38
Social Esteem	7.06	2.32	.33	−.42	.85	.83	.55	.27
Collective Self	8.30	1.64	−.22	1.32	.78	.85	.58	.37
Self-Awareness	8.70	1.77	.43	.97	.75	.76	.45	.32
Total belonging	34.64	5.87	.21	.44	.87	–	–	–

## Discussion

Measuring a sense of belonging allows psychologists and health care professionals to have a greater understanding of belonging amongst older adult clients. Measures of belonging to date have not been adapted to Persian contexts. Therefore, this study aimed to investigate the initial psychometric properties of the SOBI in older adults in Iran. Findings from the present study indicated that the Iranian version of the SOBI yielded a four-factor structure (eigenvalues > 1), which included 16 items accounting for 54.12% of the total variance. Factor loadings of these latent constructs were strong, with values ranging from .50 to .87. The findings contrast the findings from the original two factor-based scales [15]. After exploring the factor structure of the measure, a confirmatory factor analysis was conducted, which demonstrated that the four-factor latent structure of the SOBI had adequate data-model fit statistics, and all latent structures were characterized by strong latent construct reliability coefficients and adequate–to–strong internal consistency reliability (α) coefficients. The Cronbach’s alpha values were similar to those found in the original scale [15] and those found with the Thai-translated SOBI [52, 53]. Moreover, the measurement model’s reliability and validity findings indicated that the scales had adequate-to-strong composite reliability and discriminant validity. However, convergent validity results showed that the AVE scores for fitting in and social esteem were less than .50. Collective self and self-awareness structures were provided criterion of convergent validity, and the AVE scores for both scales were greater than .50, which provides initial support for the efficacy of the Persian version of the SOBI as a reliable and valid measure for measuring a sense of belonging in older adults living independently in the community.

### Limitations and future research

When interpreting the results of this study, the characteristics of the study participants should be a consideration. As the study of the older Iranian adults was a convenience sample, and the participants were living independently, results cannot be considered representative of those that may be found for the population of all Iranian adults over 60 years. The lived experience of older Iranians in residential care would likely differ from those residing in the community. Future research should consider evaluating the SOBI with older adults living in residential care, especially given the risk for loneliness in this demographic group. Additional validity of other belonging measures requires the support of the discriminant and convergent validity of the Persian form of the SOBI. In terms of preventative care, it is plausible to assume that the early identification of low belonging in younger age groups with an appropriate measure may help prevent loneliness in older adulthood, assuming the appropriate interventions can be implemented. Finally, we conducted CFA and EFA within the same sample, which is considered an additional limitation of the present study. There is a need to confirm the factor structure of the SOBI using CFA with different samples in future studies.

## Conclusions

The current study evaluated the psychometric properties of a Persian version of the SOBI. The findings indicated that the four-factor Persian version of the SOBI is a valid and reliable tool for measuring belonging in older adults over 60 living independently. The findings of this study have implications for how belonging is measured in older Iranian adults. Pending further investigation into its psychometric properties within the Iranian context, The Persian version of the SOBI offers the promise of utility for psychologists and health care professionals working with older adults living in the community.

## Data Availability

Not applicable.

## Abbreviations

SOBI: Sense of Belonging Instrument
SOBI-P: Sense of Belonging Instrument (psychological state)
SOBI-A: Sense of Belonging Instrument (antecedents)
KMO: Kaiser–Meyer–Olkin
EFA: Exploratory factor analyses
CFA: Confirmatory factor analysis
RMSEA: Root mean square error of approximation
CFI: Comparative fit index
NNFI: Non-normed fit index
SRMR: Standard root mean square residual
CR: Composite reliability
AVE: Variance extracted
MSV: Maximum shared variance
